# The Human Vaginal Bacterial Biota and Bacterial Vaginosis

**DOI:** 10.1155/2008/750479

**Published:** 2009-02-16

**Authors:** Sujatha Srinivasan, David N. Fredricks

**Affiliations:** ^1^Vaccine and Infectious Disease Institute, Fred Hutchinson Cancer Research Center, Seattle, WA 98109, USA; ^2^Division of Allergy and Infectious Diseases, Department of Medicine, University of Washington, Seattle, WA 98195, USA

## Abstract

The bacterial biota of the human vagina can have a profound impact on the health of women and their neonates. Changes in the vaginal microbiota have been associated with several adverse health outcomes including premature birth, pelvic inflammatory disease, and acquisition of HIV infection. Cultivation-independent molecular methods have provided new insights regarding bacterial diversity in this important niche, particularly in women with the common condition bacterial vaginosis (BV). PCR methods have shown that women with BV have complex communities of vaginal bacteria that include many fastidious species, particularly from the phyla Bacteroidetes and Actinobacteria. Healthy women are mostly colonized with lactobacilli such as *Lactobacillus crispatus*, *Lactobacillus jensenii*, and *Lactobacillus iners*, though a variety of other bacteria may be present. The microbiology of BV is heterogeneous. The presence of *Gardnerella vaginalis* and *Atopobium vaginae* coating the vaginal epithelium in some subjects with BV suggests that biofilms may contribute to this condition.

## 1. INTRODUCTION

The vagina is the Rodney Dangerfield of the human body; it gets no respect. Although frequently regarded as a mere passageway for menses, sperm, or neonates, the human vagina is a highly versatile organ that can profoundly affect the health of women and their newborn infants. The environment in the vagina can impact the probability of conception, the ability to carry a fetus to term, and the risk of acquiring sexually transmitted diseases such as HIV infection. Microbes play a critical role in determining the biochemical and inflammatory profile of the vaginal environment. Although decades of studies based on cultivation technologies have illuminated the microbiota of the human vagina, recent studies employing cultivation-independent methods have significantly increased our understanding of bacterial diversity in this important niche. This review will focus on the bacterial biota in the human vagina, with particular attention paid to studies using nucleic acid sequence-based approaches. We will highlight the changes in vaginal bacterial communities that are associated with the common condition bacterial vaginosis (BV) and will discuss the challenges to using Koch's postulates [1, 2] to assess evidence of causation for fastidious bacteria in these microbial communities. There are many important pathogens in the vaginal niche such as *Neiserria gonorrhea*, *Ureaplasma* species, *Mycoplasma genitalium*, *Streptococcus* species, *Escherichia coli*, *Chlamydia trachomatis*, and *Trichomonas vaginalis* which we will not explore in this review. Studies of fungal, viral, archaeal, and protistan diversity in the human vagina are important but will not be the focus of this review due to the paucity of published molecular surveys. Studies of the human vaginal microbiome in 2009 are in their infancy. Both metagenomic and whole bacterial genome sequencing projects are underway to help define the collection of microbial genes present in the vagina and to understand their contribution to normal host physiology and disease.

The picture that emerges from most studies of the vaginal microbiota described here is static because it is based on cross-sectional studies that assess the microbial constituents at discrete and infrequent time points. However, microbial communities in the human vagina likely undergo shifts in the representation and abundance of key species over time that are influenced by factors which may include age of the woman, hormonal fluctuations (e.g., stage of menstrual cycle, contraception), sexual activity (e.g., types of sexual activities such as oral or anal sex followed by vaginal sex, frequency of sex, number of sex partners, and the genitourinary tract microbiota of these partners), underlying health conditions (e.g., diabetes, urinary tract infection), use of medications (e.g., intravaginal and systemic antibiotics), intravaginal washing practices and hygiene. Future studies will benefit from the use of high throughput technologies that will facilitate measuring fluctuations in the human vaginal microbiota over time in longitudinal analyses with more frequent sampling. Current data suggest that these studies will reveal a highly dynamic human vaginal ecosystem in many women.

## 2. THE VAGINAL MICROBIOTA: “NORMAL” VERSUS BACTERIAL VAGINOSIS

**Figure 1:**
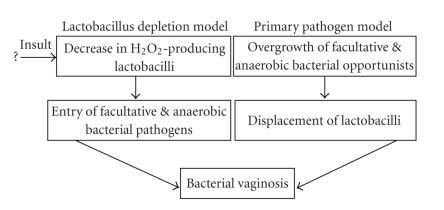
*Competing models for the pathogenesis of BV.* At least 2 models exist to explain the pathogenesis of BV. The lactobacillus depletion model suggests that there is a decrease in hydrogen peroxide producing lactobacilli as the primary event that allows for the overgrowth of facultative anaerobes resulting in BV. The primary pathogen model suggests that the entry of facultative anaerobes causes the displacement of lactobacilli thereby resulting in BV.

Gram stains of vaginal fluid smears from women without BV typically show Gram-positive rods, with cultures revealing a predominance of lactobacilli, particularly *Lactobacillus crispatus* and *Lactobacillus jensenii* [3–5]. Lactobacilli are believed to promote a healthy ecosystem by producing lactic acid, hydrogen peroxide, and bacteriocins that have antimicrobial properties thereby excluding pathogens from this niche [6]. *Lactobacillus iners* is an underappreciated member of the normal vaginal biota, as it does not grow on Rugosa agar that is typically used to isolate lactobacilli [3]. In contrast, women with the condition bacterial vaginosis (BV) have loss of many *Lactobacillus* species (except *L. iners*) and acquisition of a variety of anaerobic and facultative bacteria [7, 8]. Gram stains of vaginal fluid from women with BV show loss of Gram-positive rods and their replacement with Gram-negative and Gram-variable cocci and rods [9]. Cultures of vaginal fluid from subjects with BV typically yield *Gardnerella vaginalis* and a mixture of other bacteria that may include *Prevotella, Porphyromonas, Mobiluncus*, and *Mycoplasma* species. It is not known whether the primary event initiating BV is the loss of key lactobacilli or acquisition of the complex bacterial communities found in this syndrome; these may be simultaneous processes (Figure 1). It is also possible that some other factor is the primary etiological agent, and that the changes in vaginal microbiota reflect a downstream event in the pathogenesis of BV.

## 3. BACTERIAL VAGINOSIS

BV is the most common cause of vaginal discharge and a frequent reason for women to seek medical attention [10]. BV is highly prevalent, affecting ∼10–30% of women in the United States [11], with higher rates reported in African American women and women from Sub-Saharan Africa [12–14]. Although BV is an important medical condition itself, it is associated with several more serious adverse outcomes including preterm birth [15], pelvic inflammatory disease [16], and acquisition of HIV infection [17]. Women with BV may have a malodorous vaginal discharge or local irritation, but about half of the women with diagnosable BV have no clear symptoms [18]. Some women do not report abnormal vaginal discharge, but discharge is nonetheless noted on examination by a clinician, highlighting that many women with BV are not aware of their diagnosis or consider their discharge to be within normal bounds. The high prevalence of BV and the lack of symptoms in a substantial fraction of affected women lead to the question whether BV should be considered a normal variant of the vaginal microbiota or a disease entity. For women affected by severe symptomatic BV as manifested by profuse vaginal discharge and less frequently by local burning or itching, there is little question that they have a disease. For women with laboratory evidence of BV but no symptoms, the disease designation seems inappropriate, though the condition may still impart increased risk of adverse health outcomes such as preterm birth. Antibiotics such as metronidazole and clindamycin are usually effective in treating BV in most subjects, leading to resolution of symptoms, though rates of relapse are high [19, 20]. Either systemic (usually oral) or intravaginal antibiotics can be used to treat BV.

Symptomatic BV can be described as a syndrome based on the presence of a collection of clinical features without a specific etiologic agent defined. The diagnosis of BV is usually made using a series of clinical criteria collected by a clinician performing a pelvic examination, or by interpretation of vaginal fluid Gram stains. Amsel clinical criteria are usually employed for the diagnosis of BV in the clinical setting because the approach is rapid, but it does require access to a microscope [18]. At least 3 of 4 Amsel criteria must be present to establish a diagnosis of BV, including (1) elevated vaginal fluid pH > 4.5; (2) a positive “whiff test” which consists of the detection of a fishy odor upon addition of 10% potassium hydroxide to a slide containing vaginal fluid; (3) the presence of clue cells (>20%) in vaginal fluid which are shed vaginal epithelial cells coated with bacteria creating indistinct borders; (4) a homogeneous, milky vaginal discharge. Note that it is possible to have a diagnosis of BV based on Amsel clinical criteria without the presence of frank vaginal discharge. Accordingly, presuming that women without vaginal discharge do not have BV is not valid, and studies of the “normal” vagina should ideally employ an objective method to assess for BV. Unfortunately there are numerous studies in the field that have claimed that BV-associated bacteria are part of the normal microbiota without having assessed for BV status, although self-reported vaginal discharge may have been absent. It is possible, indeed probable, that many BV-associated bacteria can be part of the normal human vaginal microbiota, but the failure to use consensus guidelines to define BV in the research setting is a recipe for scientific confusion that is completely avoidable with well-designed studies.

An alternative method for diagnosis of BV relies on analysis of Gram stains performed on vaginal fluid smears. This approach is most commonly employed in the research setting where Gram stains are used to classify subjects but is less well suited to the clinical setting because analysis of the vaginal fluid Gram stains requires a degree of expertise that is rarely available in real time when the clinician is faced with the decision whether to treat for BV. For better or for worse, the vaginal fluid Gram stain is considered the current diagnostic gold standard as it offers greater reproducibility and objectivity when compared with the Amsel's clinical criteria. For example, there can be variation between technicians in the evaluation of wet mounts for vaginal clue cells. Several scoring systems are used to classify vaginal smears. The method of Nugent et al. [9] assesses the presence and relative amounts of three bacterial morphotypes, including Gram-positive rods (lactobacilli), Gram-negative and Gram-variable rods (*Gardnerella vaginalis*, and *Bacteroides* species), and curved rods (*Mobiluncus* species). A Nugent score of 0–3 is considered normal (no BV) and is marked by the presence of Gram-positive rods, or at least no *Gardnerella vaginalis* or *Mobiluncus* morphotypes. A Nugent score of 7–10 confers the diagnosis of BV and is marked by the absence of Gram-positive rods and the presence of high concentrations of *Gardnerella* or *Mobiluncus* morphotypes. A Nugent score of 4–6 is designated intermediate flora and has Gram stain features between the two poles. Alternative scoring systems for interpretation of vaginal fluid Gram stains exist, such as that of Ison and Hay [21].

## 4. THE ROLE OF *GARDNERELLA VAGINALIS* IN BV

In a sentinel paper published in 1955, Herman Gardner and Charles Dukes reported the successful isolation of a novel bacterium from subjects with the syndrome nonspecific vaginitis, now known as BV. The bacterium was initially named *Haemophilus vaginalis* but was later renamed *Gardnerella vaginalis*. The authors stated, “We are prepared to present evidence that the vast majority of so-called “nonspecific” bacterial vaginitides constitute a specific infectious entity caused by a single etiological agent [22].” These investigators believed that *G. vaginalis* was the sole cause of BV and set out to fulfill Koch's postulates for disease causation in a series of clinical experiments. Pure cultures of *G. vaginalis* were inoculated into the vaginas of 13 healthy women, which resulted in the development of BV in 1 of the 13, with a corresponding rate of disease production of 7.7%. Based on these data, the investigators concluded that Koch's postulates were fulfilled, though the 92% failure rate calls this conclusion into question. The investigators went on to perform an additional experiment wherein whole vaginal fluid obtained from subjects with BV was used to inoculate the vaginas of 15 women without BV. Eleven of these 15 subjects developed BV, yielding a disease induction rate of 73%. The authors felt that these data further supported the causal role of *G. vaginalis* in BV because this bacterium was cultured from most of the induced cases. It is our interpretation of these studies that whole vaginal fluid is a much more successful inoculum for the transmission of BV than is a pure culture of *G. vaginalis*, suggesting that there are other factors besides *G. vaginalis* important in disease induction.

Other evidence suggests that *Gardnerella vaginalis* is not the sole etiological agent in BV. Koch's postulates demand that the etiological microbe should be found in every case of disease but should not be detected in subjects without disease [1] (see section on Koch's postulates). *G. vaginalis* fails this later test of specificity because it can be detected in about 30–50% of women without BV using cultivation methods and 70% of women without BV using PCR methods [23]. After more than half a century, we are still debating the role of *G. vaginalis* in BV. Although *G. vaginalis* likely plays an important role in the pathogenesis of BV, it is unlikely to be the sole instigator because it is never found as the sole bacterium in vaginal fluid from subjects with BV. Our hypothesis is that BV is a syndrome caused by communities of bacteria that include uncultivated species, precluding the formal application of Koch's postulates and necessitating new approaches for establishing causation.

## 5. VAGINAL MICROBIAL DIVERSITY: THE PERSPECTIVE FROM CULTIVATION

With the advent of molecular techniques used to measure bacterial diversity, it is easy to discount the contributions from studies based on cultivation because these studies may fail to detect a large number of fastidious microbes in any given niche. However, cultivation studies provide critical insights about the phenotypic characteristics of microbes that are not easily derived from molecular studies. Furthermore, cultivated microbes allow for the experimental manipulation of these organisms in the laboratory and the testing of hypotheses about pathogenesis and virulence factors. Accordingly, cultivation studies remain an important area of investigation in vaginal microbiology, despite the limitations of the approach [24]. One reason for pursuing the combined approach using cultivation and cultivation-independent methods is that some bacteria are more likely to be detected by cultivation when present in low concentrations. For example, Verhelst et al. [25] reported that of the 38 vaginal bacterial species identified from 8 subjects with and without BV, 5 were detected by cultivation alone. Novel cultivation approaches may be required to grow the many fastidious bacterial species found in the human vagina.

Prior to Burton and Reid's study in 2002 [26], almost all of our knowledge about the bacteria in the vaginal niche came from cultivation studies which involved isolating the organisms by culture on selective or nonselective media and subsequent identification by phenotypic techniques. Just as use of a variety of broad range bacterial PCR primers helps to maximize species diversity (see section on Molecular Approaches), a number of media and growth conditions may be needed for the optimal isolation of diverse bacterial species. Relatively nonselective media such as MacConkey agar, mannitol salt agar, and tryptic soy base with 5% sheep blood agar can be useful to estimate numbers of aerobic and anaerobic bacteria in vaginal samples. Selective or semiselective media include Rogosa [27] or de Man, Rogosa and Sharpe media (MRS) for lactobacilli and the human bilayer Tween (HBT) agar for the isolation of *Gardnerella* *vaginalis* [28]. It should be noted that *Lactobacillus* *iners*, present in many subjects with and without BV, does not grow on Rogosa agar but can grow on HBT agar.

Cultivation-based approaches have identified *Gardnerella* *vaginalis*, anaerobic bacteria such as *Prevotella*, *Porphyromonas*, *Peptostreptococcus*, *Mobiluncus*, and *Mycoplasma* to be largely associated with the disturbed microbiota in subjects with BV. Healthy women are commonly colonized with hydrogen peroxide producing lactobacilli which are thought to inhibit the growth of the fastidious anaerobes associated with BV. Specific details of cultivation studies will not be discussed further but can be obtained from recent reviews [29, 30].

## 6. VAGINAL MICROBIAL DIVERSITY: THE MOLECULAR PERSPECTIVE

Cultivation-independent approaches have consistently documented the high proportion of fastidious bacteria in a variety of ecological niches [31] and these tools have recently been applied to study the vaginal ecosystem. Results from many different research groups confirm that the human vagina hosts numerous bacterial species that are either not cultivated or not easily identified using cultivation methods. These results help to augment, but do not replace, the census data generated using cultivation-based approaches. Indeed every method for characterizing the human indigenous microbiota is subject to some degree of bias. Therefore, it is our position that the most complete picture of the human microbiota will emerge from the application and synthesis of different technologies and approaches, including cultivation. We highlight both the strengths and limitations of various molecular approaches for describing the vaginal microbiota below.

The most commonly employed target for molecular identification of bacteria is the small ribosomal subunit or 16S rRNA gene. The 16S rRNA gene is useful because it is present in all bacteria and has regions of sequence conservation that can be targeted with broad range PCR primers and areas of sequence heterogeneity that can be used to identify bacteria or infer phylogenetic relationships (see [32–36]). Once the 16S rRNA gene has been sequenced from a bacterium, the variable regions can be used for species-specific PCR either in a qualitative or quantitative manner. Quantitative PCR is especially useful for rapidly identifying bacteria when an internal probe is employed and for measuring how levels of vaginal bacteria change. Nine highly variable and therefore phylogenetically rich regions of the ∼1540 base pair 16S rRNA gene have been described and designated V1 to V9 [37]. The choice of primers targeting the conserved regions flanking the different variable regions can profoundly affect the diversity of bacterial species identified [38, 39].

It is theoretically possible to detect every known bacterial species if suitable broad range PCR primers or combinations of different primer pairs are employed. Current studies focus on the extraction of total genomic DNA from vaginal fluid on swabs or from cervicovaginal lavage fluid and amplification of 16S rRNA genes with primers that bind to conserved sites present in many species. The sequences obtained are aligned and compared to large databases of 16S rRNA sequences (http://greengenes.lbl.gov/ [40], http://rdp.cme.msu.edu/ [41, 42], http://www.arb-home.de/ [43] to infer phylogenetic relationships to known species. Some studies rely on the construction of clone libraries and direct sequencing of a particular number of clones [44, 45]. This approach allows for good phylogenetic resolution if a suitable portion of the 16S rRNA gene is amplified. However, this method tends to be expensive, slow, and tedious. Some investigators try to limit the sequencing of large numbers of samples by using electrophoretic fingerprinting techniques such as denaturing gradient gel electrophoresis (DGGE) [46] or terminal restriction fragment length polymorphism (T-RFLP) [47]. In the case of DGGE, as the amplification products pass through the denaturing gel, their melting behavior depends primarily on the length of the product and the GC content [48]. Typically one of the primers carries a 5′-GC rich clamp, around 40 bp, which is used to detect single-base changes between close products. This clamp tends to lower the PCR amplification efficiency and can increase the presence of PCR artifacts such as heteroduplexes [48]. T-RFLP involves PCR amplification of the community DNA using primers with fluorescent tags. The resulting PCR products are digested with restriction enzymes and the fluorescent terminal restriction products are detected using a DNA sequencer. The species diversity revealed by DGGE is much less than the diversity detected by T-RFLP [49], and this likely reflects greater sensitivity of the fluorescence detection platform. Screening clones in a library by amplified ribosomal DNA restriction analysis (ARDRA) in order to limit the number of clones to be sequenced is also commonly used. ARDRA is based on the restriction digestion of 16S rRNA gene clones or amplified DNA and electrophoretic separation on high percent agarose or polyacrylamide gels [7].

An approach complementing broad range PCR is characterization of the vaginal bacterial community by using nucleic acid probes, oligonucleotides complementary to rRNA gene targets. Probes are designed using sequences generated from broad range PCR and sequencing experiments which can have a wide range of phylogenetic specificities ranging from domain to strain levels. There is also a database maintaining probes designed for many bacteria from other niches (http://www.microbial-ecology.net/probebase/) [50]. The probes are labeled with a fluorescent tag and hybridized to the clinical samples. Cells are visualized using epifluorescence microscopy in a process referred to as fluorescence in situ hybridization (FISH). Data can be collected in both quantitative and qualitative modes. For example, when fluorescent probes are combined with flow cytometry, one can rapidly count and collect cells. With confocal scanning laser microscopy, one can visualize the spatial arrangement of cells in tissues or body fluids.

## 7. DIVERSITY STUDIES BASED ON THE 16S rRNA GENE: LIMITATIONS

While molecular methods have many advantages over cultivation approaches for characterizing microbial diversity, there are numerous limitations [51–53]. Use of some so-called “universal primers” targeting conserved regions of the 16S rRNA gene may not detect all bacteria present in a sample due to the presence of polymorphic nucleotides at conserved positions. The primers are more accurately designated as broad range. Heterogeneity of the 16S rRNA gene within the same species can also hamper fingerprinting analysis [39]. Lowering the annealing temperature during PCR permits mismatches when using broad range primers thereby increasing the diversity of the PCR products formed, though this may also allow nonspecific amplification of DNA from human tissues. Degenerate nucleotides can help in overcoming the deficiency of broad range primers when polymorphic base positions are encountered but can lead to lower efficiency of primer binding due to exact matches of variants being diluted in the primer pool. If the primer concentrations are increased to overcome this dilution problem, then there is the potential for increased nonspecific product formation. Inosine-based primers are an alternative to degenerate primers [54] but these cannot be successfully used with *Pfu* [55], a high fidelity polymerase. One example of a commonly used broad range PCR primer targeting the 16S rRNA gene is the 27f (8f) primer at the 5′ end of the 16S rRNA gene. This primer has multiple mismatches with many Chlamydiae and Bifidobacteria, highlighting the fact that this primer may be highly inefficient in detecting bacteria in these phylogenetic groups [38]. More frequently, individual species within phylogenetic groups may have mismatches that result in reduced amplification efficiencies [30]. Frank et al. [38] evaluated the 27f (8f) primers (designated as 27f-CC and 27f-CM) that are commonly used in many broad range PCR studies and formulated a 27f primer mixture (designated as 27f-YM+3) that included three sequences not usually accounted for in many contemporary studies. These primers are better matches with bacteria in the *Chlamydiales* and *Bifidobacteriales* orders as well as bacteria in the *Borrelia* genus. Using a combination of linear amplification with the 27f formulation and quantitative PCR, they showed that the formulated primer mixture performed better at detecting *Gardnerella vaginalis* sequences even at elevated annealing temperatures (60°C) than the 27f primers typically used in the literature. Several studies have attempted to characterize the vaginal bacterial biota using the conventional 27f primer and these studies appear to underrepresent bacteria such as *G. vaginalis* which is a common member of the vaginal ecosystem [25, 56]. Although use of complex primer mixtures may increase the diversity of bacteria detected by broad range PCR, this advantage comes at a cost. When using the primer mixture, there is a slight decrease in amplification efficiency due to a reduction in primer concentrations with exact matches.

**Figure 2:**
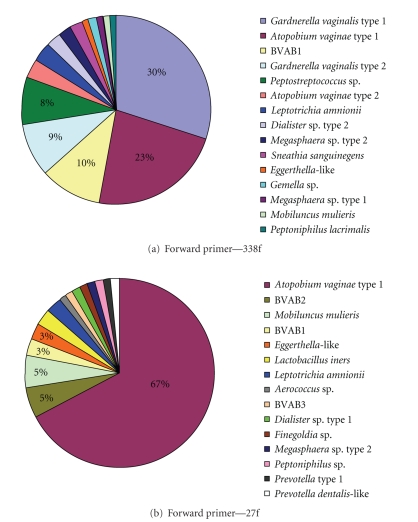
*Comparison of vaginal bacterial species detected by broad range 16S rRNA gene PCR using two different forward primers and the same reverse primer in one sample.* The pie charts show the percentages of clones in each library corresponding to specific bacterial 16S rRNA gene sequences obtained using broad range PCR followed by cloning and sequencing in a vaginal sample from a subject diagnosed with bacterial vaginosis. Data obtained using the 338f (a) primer shows a balanced representation of clones while the data obtained using the 27f (b) primer is skewed toward *Atopobium vaginae*. Note the absence of *Gardnerella vaginalis* clones in the clone library created with the 27f primer. BVAB denotes bacterial vaginosis associated bacterium.

We have seen similar problems with the 27f primer in our broad range bacterial PCR studies of the vaginal niche. We amplified a region of the ribosomal RNA operon using 27f [57] modified with one degeneracy (27f-CM) and 189r [58] at an annealing temperature of 55°C. Clone library analysis on a model subject with BV revealed the absence of *Gardnerella vaginalis* (Figure 2, unpublished data). In contrast, by utilizing a different forward primer (338f) and the same reverse primer, *G. vaginalis* emerged as the dominant clone in the library (Figure 2). Moreover, as can be seen in Figure 2, use of different forward primers on the same vaginal sample results in vastly differing rank abundance plots. For example, a fastidious bacterium in the *Clostridiales* order designated BV-associated bacterium 1 (BVAB1) was detected using the 338f primer while all three novel bacteria in the *Clostridiales* order associated with BV (BVAB1, BVAB2, BVAB3) were detected with the 27f primer. The 5 most prevalent clones detected with 338f included sequences matching *G. vaginalis* type 1, *Atopobium vaginae* type 1, BVAB1, *G. vaginalis* type 2, and *Peptostreptococcus* while the most abundant clones seen with the 27f primer were *A. vaginae* type 2, BVAB2, *Mobiluncus mulieris*, BVAB1, and an *Eggerthella-*like bacterium. Using both sets of primers, we detected a total of 22 phylotypes of which 10 were represented as singleton species (detected as a single clone). When this is compared with each primer pair alone, we were able to detect only 15 phylotypes each, including 5 singletons with the 338f primer and 8 single clones with the 27f primer. We also noted that the 27f primer in combination with 189r tended to be biased to *A. vaginae*, thereby not providing representative reflections of bacterial abundance (Figure 2). However, we found that creating two clone libraries with different forward primers resulted in detection of more phylotypes, again highlighting the limitations imposed by the selection of a single primer pair. Accordingly, we suggest that using combinations of broad range primers on the same sample may maximize the diversity of species detected, though this comes at a cost of additional time and money expended.

The DNA extraction step is vital to getting a representative pool of DNA which will then be used for PCR amplification. Species bias for different extraction methods is well known [59, 60]. Presence of inhibitors in the clinical samples from blood, mucus, or vaginal products can lead to failed amplification or a reduction in the amount of product. Amplification controls are useful in tracking DNA quality wherein PCR of specific target genes such as beta-globin [23] or the 18S rRNA gene [61] can indicate if the DNA extracted from human tissues is amplifiable. Use of internal amplification controls by adding an exogenous template at known concentrations to the clinical samples can help in detection of subtle PCR inhibitors [62, 63], particularly when performing quantitative PCR analysis.

Another issue with broad range PCR targeting the 16S rRNA gene is the lack of phylogenetic resolution for some bacteria, even at the species level. For example, different species within the *Enterobacteriaceae* have very similar 16S rRNA gene sequences. Other gene targets offer improved phylogenetic resolution for some species, such as the sigma factor *rpoB* present in just one copy per genome [64–67]. A downside of using *rpoB* as a marker is the dearth of sequences available when compared to the 16S rRNA gene. An alternate option is to examine the internal transcribed spacer region by ribosomal intergenic spacer analysis to distinguish closely related strains [68–70]. Here again, sequence and size heterogeneity can be critical limitations, and databases (http://egg.umh.es/rissc/) supporting this region are small in comparison to those supporting 16S rRNA gene sequences.

Correlating the number of 16S rRNA gene copies (and hence clones) to the number of bacteria is frequently not possible as different bacterial species can have varying numbers of rRNA gene operons per genome (between 1 and 15) and the exact number is unknown for most species [71–73]. Bacteria with higher rRNA operon copy numbers will be excessively represented in a clone library when compared with bacteria with lower copy numbers, thereby introducing a bias in the community analysis [74]. Moreover, different bacteria may have varying susceptibilities to lysis based on the extraction methods being used thus leading to different quantities of bacteria observed in subsequent analysis.

Similarly, false positives can impact community analysis when targeting the 16S rRNA gene using broad range primers. Low levels of bacterial DNA may be present in laboratory or PCR reagents and in DNA extraction kits. *Taq* polymerase used for PCR amplification can have contaminating 16S rRNA sequences [75, 76]. A way to monitor this problem is to include negative controls in every run of PCR. No template PCR controls allow for detection of contaminants arising from PCR reagents and the water being used in every PCR experiment. Additionally, it is extremely useful to include extraction controls wherein sham samples are processed and extracted in the same manner as the experimental samples. These extraction controls should be subjected to PCR and analysis of products (such as cloning/sequencing) alongside samples of interest to identify any contaminants. Limiting the number of amplification cycles and using high amounts of template DNA also help in reducing amplification of low level contaminants that may have been introduced during the different steps of sample preparation. An important source of PCR contamination is from previously amplified products. This can be managed by separating pre- and post-PCR working spaces, use of aerosol filter pipette tips, and addition of uracil glycosylase to inactivate previously amplified PCR products.

The PCR amplification step itself can introduce biases such as skewed representation of a sample based on the guanosine plus cytosine (G+C) content of the bacterium [77, 78]. Bacteria with higher G+C content may result in lower throughputs when compared with bacteria with lower G+C. PCR enhancing additives such as betaine [79], dimethyl sulfoxide [80], or formamide [81] are typically used to equalize the read-through efficiencies of the different templates with varying G+C contents while the reducing environments created by *β*-mercaptoethanol or dithiothreitol [82] seem to provide unspecified PCR enhancing effects. PCR enhancers that are commercially available (e.g., Q-solution from Qiagen, PCR enhancer solution from Invitrogen) can be expensive and their composition is not known. Low cost in-house reagents such as a combination of betaine, dithiothreitol and dimethyl sulfoxide have been shown to improve both qualitative and quantitative outputs of PCRs [83].

PCR artifacts are a well-known limitation when using the broad range PCR approach. Incorporation of incorrect nucleotides using *Taq* polymersase may lead to errors in the sequence. Heteroduplexes may form when primers become limiting and/or there is greater template diversity [84, 85]. Use of *Pfu* polymerase which possesses 3′ to 5′ exonuclease proofreading capabilities allows for the correction of misincorporated nucleotides and hence has fewer errors when compared with *Taq* polymerase [86]. There are other high fidelity DNA polymerases that are currently available such as Vent DNA polymerase isolated from *Thermococcus litoralis* and Phusion DNA polymerase which is a *Pyrococcus*-like enzyme with a double-stranded DNA-binding domain. One recommended strategy to limit heteroduplex molecules prior to cloning is to reamplify 10-fold diluted PCR product containing mixed templates in a process referred to as “reconditioning PCR” [85]. Formation of chimeras [87, 88] needs careful monitoring and identification. Chimeric sequences are PCR artifacts that arise when two or more phylogenetically distinct sequences become combined into a single sequence when the polymerase jumps between templates during extension. Several online tools are available to detect chimeras such as Bellerophon [89], Mallard [90], or Pintail analysis [91].

While the broad range 16S rRNA gene PCR approach provides a good census of the bacteria present in the clinical sample, no functional genomic information is obtained. Metagenomic approaches have been applied to environmental samples [92, 93] but are slow to be applied to the vaginal environment due to lack of whole genome sequence information for creation of a scaffold. There is presently an NIH-led initiative to sequence whole genomes from cultivable bacteria from the vaginal niche which will provide the necessary foundation for metagenomic studies (http://nihroadmap.nih.gov/hmp/).

## 8. MOLECULAR STUDIES IN THE VAGINAL NICHE: A CRITICAL EXAMINATION

With advancing technologies and decreasing costs of sequencing, there have been many recent additions to our knowledge regarding the human vaginal microbiota. As conditions in the vagina may be transient and dependent on numerous factors, most molecular studies offer a snapshot of the vaginal microbiota under specific conditions. Moreover, with differing definitions of “normal,” it can be difficult to compare the data across many studies. We present here a survey of key molecular investigations in the vaginal niche, highlighting the important contributions and the limitations of each approach.

Burton and Reid [26] were the first investigators to analyze the microbiota of the vaginal niche using broad range molecular methods. They applied a combination of broad range bacterial PCR using primers HDA-1-GC (338f with a GC clamp) and HDA-2 (515r) and DGGE to vaginal samples obtained from 20 asymptomatic postmenopausal women and used Nugent scores to distinguish between healthy and diseased states. Interestingly, 70% of the women had intermediate flora or BV as indicated by Nugent score, suggesting that women with abnormal vaginal flora were overrepresented in their study compared to the general population. Broad range PCR targeting about 200 bp of the V2-V3 variable regions of the 16S rRNA gene and DGGE analysis showed that subjects with low Nugent scores had only one to two bands, mainly derived from *Lactobacillus* species, while subjects with intermediate flora or high Nugent scores had zero to four bands representing *Gardnerella*, *Prevotella*, *Peptostreptococcus*, *Bacteroides*, *Lactobacillus*, *Streptococcus*, and *Slackia* species. The detection of *Lactobacillus iners* in subjects with normal flora by Gram stain was a novel finding. Genus specific PCR was also used to monitor the bacterial species detected by broad range PCR. The strength of this study is the utilization of both broad range and taxon-specific PCR approaches, but the DGGE method may have limited the diversity detected.

**Figure 3:**
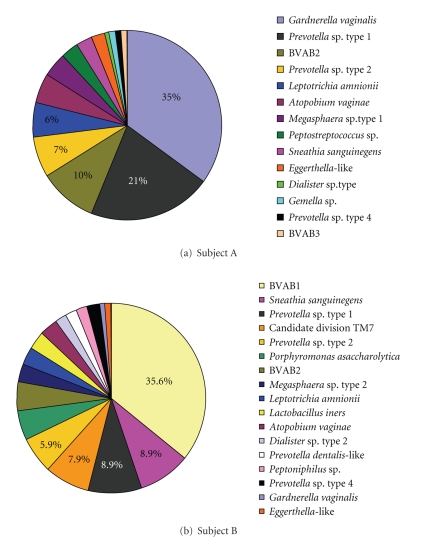
*The microbiology of BV is heterogeneous.* Comparison of rank abundance plots from 2 subjects diagnosed with BV. The charts show the percentages of clones in each library corresponding to specific bacterial 16S rRNA gene sequences obtained using broad range PCR followed by cloning and sequencing. The most prevalent bacterial clones in Subject A include those matching *Gardnerella vaginalis*, *Prevotella* sp. type 1, BVAB2, *Prevotella* sp. type 2, and *Leptotrichia amnionii*. In contrast, the most prevalent clones in Subject B include BVAB1, *Sneathia sanguinegens*, *Prevotella* sp. type 1, candidate division TM7, and *Prevotella* sp. type 2.

An important observation from this study [26] is that different subjects with BV had different DGGE profiles indicating heterogeneity in the composition of bacterial taxa in subjects with BV. We have observed similar results using different methods. For example, Figure 3 illustrates the differences observed in the composition and number of bacterial phylotypes in two subjects with BV. Vaginal samples were subjected to broad range 16S rRNA gene PCR using primers 338f and 1407r followed by cloning, sequencing, alignment, and phylogenetic analysis. The most prevalent phylotypes in Subject A include *Gardnerella vaginalis*, *Prevotella* sp. type 1, BVAB2, *Prevotella* sp. type 2, and *Leptotrichia amnionii*. In contrast, the most prevalent bacterial clones in Subject B include BVAB1, *Sneathia sanguinegens*, *Prevotella* sp. type 1, Candidate division TM7, and *Prevotella* sp. type 2, thereby illustrating the differences in bacterial phylotypes between two subjects with BV.

In a subsequent study from these investigators, the same primers HDA-1-GC and HDA-2 with the same PCR conditions were applied to 6 samples obtained weekly from a 51-year-old woman with recurrent BV (determined by Nugent score). Overall, 7 bacterial species were detected including *Klebsiella oxytoca, Serratia fonticola, Citrobacter freundii, Morganella morganii, Kluyvera ascorbata, Escherichia coli*, and *Staphylococcus epidermidis* [94]. None of the bacteria typically associated with the vaginal niche were detected in this study. Similarly, when the primers HDA-1-GC and HDA-2 were applied to vaginal samples from a cohort of 34 HIV-seronegative Nigerian women with BV, atypical BV-associated bacteria were detected by broad range PCR and DGGE [95]. Surprisingly, of the 34 samples, 10 had only 4 bands, 16 had 3 bands, 6 had 2 bands, and 2 had one band. If each band corresponds to a single bacterial phylotype, the bacterial diversity associated with BV in this study is substantially lower than the diversity detected in other studies and likely reflects the limits of the DGGE method employed. The dominant organism in 35% of subjects was found to be *Mycoplasma hominis.* An uncultured *Streptococcus* sp. was found in 24% of the subjects and a bacterium related to a rainbow trout intestinal bacterium was found in 26% of subjects. The absence of several prominent BV-associated bacteria may be related to the choice of primers, although the authors used the same primers to detect *Gardnerella*, *Prevotella*, *Mobiluncus*, and *Atopobium* sp. in a previous study [26]. The different results observed in this study could also be due to differences in annealing temperatures: 56°C in the earlier study [26] and 60°C in the later study [96], or due to differences in subject populations. The primers used in these studies have a 40-mer GC clamp that has been included for DGGE analysis resulting in primers that are 60 bases long, which may contribute to inefficient amplification. Based on the data presented, the authors suggest that the bacteria associated with BV in Nigerian women are different from those bacteria associated with BV in other populations of women studied. Additional molecular studies evaluating the bacterial community associated with BV from a variety of women representing different demographic groups are required to assess the degree of heterogeneity in vaginal microbiota among women.

Zhou et al. [45] investigated the bacterial community in 5 “apparently healthy” women. The women were classified as healthy using a combination of gynecological exams and self-reported symptoms, but data on Amsel's clinical criteria or vaginal fluid Gram stains were not obtained or provided. This is a major limitation of this study as many women with BV are asymptomatic. A 920 bp fragment of the 16S rRNA gene was amplified using primers 8f, also known as 27f (actual primer sequence not specified), and 926r and the products were cloned and sequenced. Between 176 and 250 clones were sequenced from each subject resulting in 2 to 7 bacterial phylotypes per subject. Two subjects had vaginal bacterial biotas dominated by *Lactobacillus crispatus,* while *Lactobacillus iners* was detected in 3 subjects. These investigators suggest that three novel taxa were associated with the healthy vagina including *Atopobium vaginae*, a *Megasphaera* species, and a *Leptotrichia* species. However, these bacteria have been associated with BV by other investigators [7, 25, 26, 97]. As standard objective criteria were not used for the diagnosis of BV, it is difficult to draw conclusions from this study about the constituents of the normal vaginal bacterial biota.

Hyman et al. [44] surveyed the bacteria on the vaginal epithelium by broad range PCR, clone library construction, and sequencing approximately 1400 bp of the 16S rRNA gene in 20 premenopausal women who were presumably healthy. While physical exams were conducted in the clinic and the women were reported to be asymptomatic, the authors did not report data on BV status using Amsel's clinical criteria or vaginal fluid Gram stains; this is a significant limitation of the study. PCR amplification of the genomic DNA was conducted using the conventional 8f (27f-CM) and 1492r primers. The forward primer has one mismatch to *Atopobium* spp. and the reverse primer also has poor homology possibly leading to poor representation of *Atopobium* spp. in the libraries. One thousand clones were selected for each subject and sequenced from both ends using conventional sequencing. Four of the 20 subjects had only *Lactobacillus* species with very high sequence diversity indicating that these vaginal bacteria were not clonal. Nine subjects had a combination of *Lactobacillus* spp. and other bacteria including *Bifidobacterium*, *Gardnerella*, and *Atopobium*. The remaining group of 7 women did not have any lactobacilli but were colonized with mixed bacterial populations that include bacteria that have been associated with BV by other investigators. This study provides a rich resource of vaginal bacterial 16S rRNA gene sequences in GenBank, but would have been more useful if additional clinical and microbiological data had been collected to exclude women with BV or define those with the condition. These investigators detected sequences from some bacteria such as *Pseudomonas* and *Stenotrophomonas* species in clone libraries that are known PCR contaminants. It would have been helpful to describe PCR and extraction controls to prove that these bacteria are arising from the vaginal epithelium and are not spuriously detected by broad range PCR.

Verhelst et al. [25] used a combination of cultivation and molecular techniques to identify vaginal bacteria in 8 subjects of whom 3 had normal flora, 2 had intermediate flora, and 3 had BV as determined by the Gram stain method of Ison and Hay [21]. Isolates from culture studies were identified using either 16S rRNA gene sequencing or by evaluating the fingerprinting patterns of the spacer regions between transfer RNA genes. Broad range PCR with primers 10f (27f-CC, not including the first two bases of the 27f primer) and 534r was used to amplify a ∼500 bp fragment of the 16S rRNA gene resulting in 854 clones from the 8 subjects. The clones were analyzed using ARDRA and clones with unique ARDRA patterns were sequenced for identification of the bacteria. A total of 38 species were identified using both approaches, of which 18 were detected by cloning only, 5 were detected by culture alone. Healthy subjects had vaginal bacterial biotas dominated by lactobacilli whereas subjects with intermediate flora or BV flora had greater bacterial diversity. *Atopobium vaginae* and several BV-associated bacteria were detected in a large number of clones generated from subjects with abnormal flora. The primers selected for broad range PCR proved to be a poor match for detecting *Gardnerella vaginalis,* which was isolated by cultivation. However, *G. vaginalis* specific PCR showed that this bacterium was associated with BV. This study underscores the importance of using a combination of approaches to attain a complete picture of vaginal bacterial diversity and the need to optimize primers for broad range PCR. The use of Gram stain analysis to evaluate BV status is commendable.

**Figure 4:**
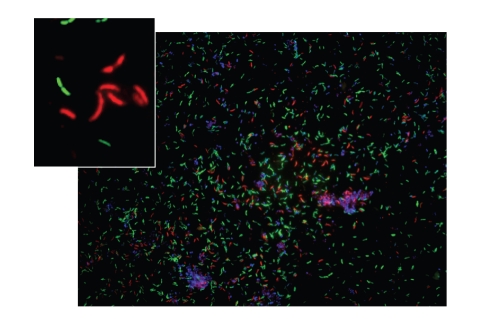
*Fluorescence image of vaginal fluid from a subject with BV.* Bacteria are shown hybridizing with probes targeting BVAB1 (green) and *Mobiluncus* (red) and visualized by fluorescence in situ hybridization (FISH). Other bacteria (blue) are seen with 4′,6-diamidine-2-phenylindole, dihydrochloride, (DAPI), which stains DNA. The inset shows that *Mobiluncus* (green) is larger than BVAB1 (red) but has the same curved morphology. (With permission from D. N. Fredricks, T. L. Fiedler, and J. M. Marrazzo, “Molecular identification of bacteria associated with bacterial vaginosis,” *New England Journal of Medicine*, vol. 353, pp. 1899–1911, 2005.)

**Figure 5:**
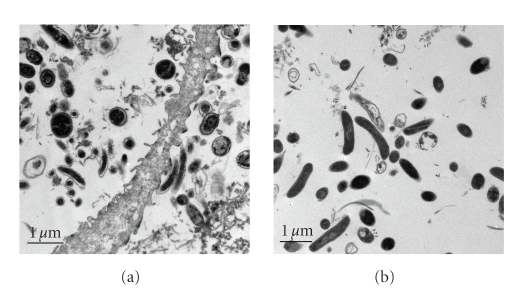
*Transmission electron micrographs.* (a) Electron micrograph of vaginal fluid from a woman with bacterial vaginosis and high concentrations of bacterial vaginosis associated bacterium 1 (BVAB1) shows many curved rods with an electron translucent zone at the outer edge of the cell. (b) These cells are different from the larger, wider, and more electron dense curved rods observed in a pure culture of *Mobiluncus curtisii*. Both images are at 20 000x magnification.

Fredricks et al. [7] evaluated the bacterial community in the vaginal niche using broad range PCR with primers 338f and 1407r amplifying a ∼1000 bp fragment from the 16S rRNA gene. This approach was applied to vaginal samples from 9 subjects with BV and 8 without BV using Amsel's clinical criteria to define BV in a cross-sectional analysis. In addition, serial vaginal samples were also obtained from a limited number of subjects to study the change in bacterial composition associated with incident, cured, relapsing, and persistent BV. One hundred clones from each subject were selected and screened using ARDRA with two restriction enzymes. Inserts with unique patterns were sequenced. Women with BV showed a high level of species diversity with a mean of 12.6 bacterial phylotypes versus women without BV who had a mean of 3.3 phylotypes per clone library. *Lactobacillus* species, particularly *Lactobacillus crispatus* and *Lactobacillus iners* were predominant in women without BV. *L. crispatus* was not detected in subjects with BV, although *L. iners* was widely prevalent. Other bacteria detected in subjects with BV included *Gardnerella vaginalis*, *Megasphaera*, *Leptotrichia*, *Dialister*, *Atopobium*, and several bacterial vaginosis associated bacteria (BVABs) from the *Clostridiales* order. Three novel bacteria from the *Clostridiales* order were highly specific indicators of BV [7]. BVAB1, BVAB2, and BVAB3 belong to the phylum Clostridium but are not closely related to any bacteria with known 16S rRNA gene sequences. A subject with incident BV had a shift from a biota dominated by lactobacilli to one with increased diversity including many putative anaerobes. A subject with cured BV had an increase in lactobacilli clones and a contraction in species diversity. A subject with relapsed BV had great diversity on day 0 with BV, followed by a contraction to predominantly *L. iners* on day 28 with cure, and then an expansion of phylogenetically rich microbiota on day 100 with relapse. A subject with persistent BV had a consistently diverse vaginal biota on days 0, 24, and 64, though there were some changes in species representation over time. A limitation of this study is that use of ARDRA to screen clones for sequencing could have underrepresented the bacterial diversity observed, as this approach tends to lump together different phylotypes with similar sequences. Moreover, only 100 clones were analyzed per library (or vaginal sample) and this limited the detection of minority species. In order to visualize the bacteria, FISH was performed on vaginal smears targeting each of the novel BVABs. BVAB1 was shown to be a thin curved rod (Figure 4); BVAB2 appears as a short, fat rod and BVAB3 is a long, lancet-shaped rod. We have performed transmission electron microscopy on a vaginal sample containing high levels of BVAB1 as determined by broad range PCR with clone library analysis, species-specific PCR, and FISH experiments. The electron micrographs show long curved bacteria with a translucent zone in the outer edge of the cells which we presume to be BVAB1 (Figure 5). This is in contrast to the larger, wider, and homogeneously electron dense cells observed in a transmission electron micrograph of *Mobiluncus curtisii* obtained from a pure culture.

**Figure 6:**
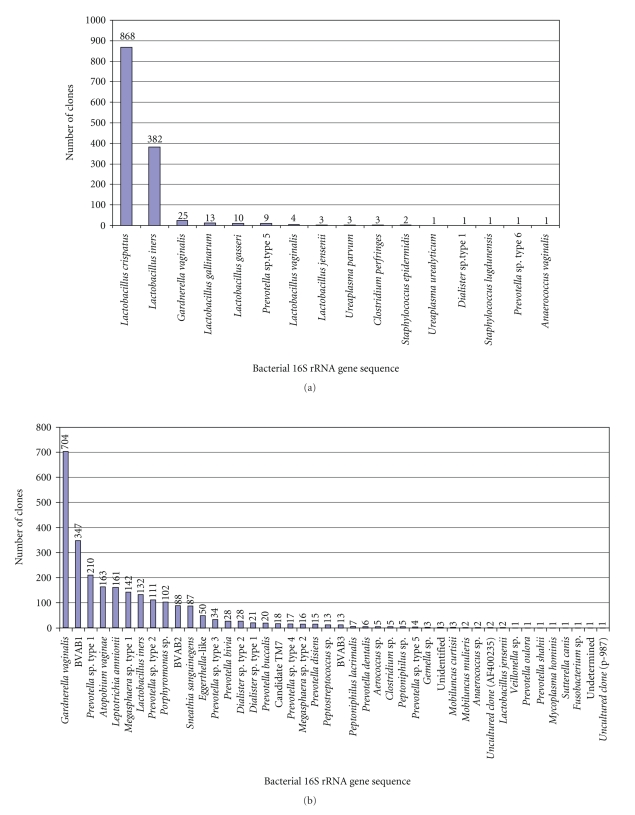
*Summary data of rank abundance plots depicting the bacterial species detected in clone libraries from subjects without BV (A) and with BV (B) in our studies.* Broad range PCR using primers 338f and 1407r along with clone library analysis of 1327 clones from 13 subjects without BV resulted in 16 phylotypes being detected. Similar analysis of 2577 clones from 23 clone libraries from 17 subjects with BV resulted in the detection of 44 different bacterial species. Vaginal bacterial species are indicated on the x-axis and the numbers of clones are indicated on the y-axis and above every bar. Subjects without BV have bacterial biotas dominated by lactobacilli while subjects with BV have a diverse bacterial biota. BVAB denotes bacterial vaginosis associated bacterium.

We have further compiled clone library data from subjects with and without BV (Figure 6). Using broad range bacterial PCR with 16S rRNA gene primers 338f and 1407r, 1327 clones were sequenced from 13 subjects without BV (Figure 6(a)). Of the 1327 clones analyzed, 65.4% of the sequences were *Lactobacillus crispatus* and 28.8% represented *Lactobacillus iners* clones. The remaining 5.8% of clones included other bacteria such as *Gardnerella vaginalis* and other lactobacilli (Figure 6(a)). These data further validate that subjects without BV have vaginal bacterial biotas dominated by lactobacilli. In contrast, analysis of 23 clone libraries from 17 subjects with BV produced 2577 clones and demonstrated a very high degree of bacterial diversity (Figure 6(b)). Each subject with BV had an average of 14 species and the top 12 phylotypes accounted for 89% of clones sequenced. The remaining 11% of sequences represented 32 phylotypes. Currently, we do not appreciate the role of the “long tail” of less prevalent bacteria though it is likely that they contribute to metabolic and functional diversity in this niche. Moreover, the diversity of bacteria observed in women with BV suggests that this may be a polymicrobial syndrome.

The use of broad range bacterial PCR combined with cloning and sequencing provides a reasonable estimate of the diversity of the most abundant bacteria but is an expensive approach with low throughput. Thies et al. [98] used a combination of broad range PCR amplification of the 16S rRNA gene in combination with T-RFLP fingerprinting to characterize the vaginal bacterial communities in vaginal swabs from 50 women with BV and 20 healthy women as determined by Nugent scoring. The authors propose that PCR combined with T-RFLP is useful to rapidly assess the most abundant bacteria and hence can be used as a tool to screen for BV. Primers for amplification included 27f (27f-CC) and 926r and were labeled at the 5′ using 6-carboxyfluorescein (6-FAM) and 4,7,2′,4′,5′,7′-hexachloro-6-carboxyfluorescein (HEX), respectively. The restriction fragment lengths were determined using an automated sequencer and the fragments were analyzed using an in-house software program. Identification of the fragments was verified by sequencing of the PCR products. A total of 23 phylotypes were detected in the samples from subjects with BV, with a mean of 6.3 phylotypes per subject (range 2–14) including *Atopobium vaginae*, *Gardnerella vaginalis*, *Megasphaera* sp., *Lactobacillus iners*, *Eggerthella* sp. and BVAB1, BVAB2, and BVAB3. Note that the species richness detected in subjects with BV in this study was less than that reported by investigators using different molecular approaches. In concordance with the results obtained in other studies [7, 44], *Mobiluncus* sp. was detected in only 2 of the 50 subjects with BV. Only lactobacilli including *Lactobacillus iners*, *Lactobacillus crispatus* group, and *Lactobacillus gasseri* group were detected in samples from subjects without BV. One of the limitations of this fingerprinting approach is the inability to distinguish between closely related species. For example, the study authors were unable to differentiate between *Mobiluncus curtisii* and *Mobiluncus mulieris* and also between the different *Prevotella* phylotypes. This resolution problem could account for the low numbers of phylotypes per subject that was observed in this study. A key strength of the study is the large number of samples processed from subjects with/without BV defined by Gram stain.

Ferris et al. [97] PCR amplified a 300 bp portion of the 16S rRNA gene with broad range primers 1055f and 1392r from vaginal samples obtained from subjects with and without BV as determined from vaginal fluid Gram stains. The DNA was subjected to DGGE and bands confirmed as *Atopobium vaginae* were identified in 12 of the 22 subjects with BV and only in 2 of the 24 control subjects. *A. vaginae* was also isolated by cultivation from 2 subjects and was shown to be metronidazole resistant. In a separate study, *A. vaginae*-specific PCR primers amplifying a 155 bp amplicon were applied to the same study cohort [99]. The specific primers further enhanced the detection of *A. vaginae* in subjects with BV while this bacterium was not detected in BV negative subjects leading to the suggestion that *A. vaginae* is highly specific for BV. PCR amplification using universal bacterial primers and T-RFLP studies also showed a correlation of *A. vaginae* to BV by Verstraelen et al. [100].

Fredricks et al. [23] used a targeted PCR approach to detect 17 key vaginal bacteria in a more sensitive fashion than is possible with broad range PCR. The PCR results were compared with the current consensus diagnostic methods for BV in order to determine if a qualitative PCR approach could be used for the molecular diagnosis of BV. Specific primers targeting various regions of the 16S rRNA gene that are specific to the bacterial species were designed. The bacteria were chosen based on clone library data previously generated [7], their apparent specificity for BV, or their novelty. All PCR products were sequenced to confirm their similarity to the intended target. The primers were applied to 264 vaginal samples obtained from 81 subjects with BV and 183 subjects without BV. Bacteria from the *Clostridiales* order, *Atopobium*, an *Eggerthella*-like bacterium, *Sneathia/Leptotrichia*, *Megasphaera* types 1 and 2, and a bacterium from the TM7 division were highly specific for BV. *Lactobacillus crispatus* was inversely associated with BV with an odds ratio of 0.02 confirming that it is largely associated with healthy vaginal flora. *Gardnerella vaginalis*, typically associated with BV, was found to have poor specificity for BV. *G. vaginalis* was found in 96% of subjects with BV but was also detected in 70% of the subjects without BV. The combination of detecting one of the *Clostridiales* bacteria (BVAB2) or *Megasphaera* type 1 produced the best sensitivity and specificity for PCR diagnosis of BV, regardless of the gold standard diagnostic criteria employed (sensitivity 99% and specificity 89%). This suggests that PCR amplification of key vaginal bacteria can indeed be used for the molecular diagnosis of BV. However, the approach used here requires electrophoresis to detect the amplification products which may not be optimal in clinical settings. A better approach would be to use quantitative PCR that offers real-time results and the ability to quantify bacteria. Levels of the bacteria may be a better indicator of disease than the presence/absence of particular species.

Some studies have investigated the utility of quantitative PCR (qPCR) as a diagnostic tool for BV. Sha et al. [101] were the first group to examine the use of qPCR for the diagnosis of BV, targeting *Gardnerella vaginalis*, *Mycoplasma hominis*, and *Lactobacillus* species using 203 samples from women with BV (Nugent score 7–10) and 203 samples from women without BV (Nugent score 0–3). Only 75 of the 203 women with BV by Nugent score were positive by Amsel criteria. Increasing levels of *G. vaginalis* and *M. hominis* and decreasing levels of lactobacilli were shown to be significantly associated with BV with a sensitivity and specificity of 83% and 78% when compared with Nugent score. The study did not evaluate women with intermediate flora.

In a subsequent study, Menard et al. [102] also investigated the association of *Gardnerella vaginalis* as well as *Atopobium vaginae* loads by quantitative PCR and assessed their utility as a diagnostic tool in 231 samples from 204 women. Nugent criteria were used to assess BV status, classifying 167 samples as normal flora, 20 samples as BV, and 44 samples as intermediate flora. They showed that the combination of the presence of *A. vaginae* at the DNA level ≥10^8^ copies/mL and *G. vaginalis* at ≥10^9^ copies/mL had a sensitivity and specificity of 95% and 99%, respectively. However, subjects with intermediate flora were excluded from this analysis. Unfortunately, the promising results from this study do not reflect how these assays would perform in a clinical setting where all women are being screened for BV, including those with intermediate flora on Nugent score. It would have been helpful to collect data on Amsel clinical criteria in these women to assess BV status using an alternative standard to determine the reliability of the molecular approach in all women. Another limitation is the relatively small number of women with BV (20) in the study. A smaller validation cohort of 56 women was assessed, of which 7 were considered to have BV by Gram stain and 10 intermediate flora. Eleven of these 56 women had molecular criteria for BV. It is not clear if the authors are proposing to treat all women with intermediate flora for BV when they have molecular evidence of BV-associated bacteria.

Zozaya-Hinchliffe et al. [103] assessed the prevalence and abundance of uncultivated *Megasphaera*-like bacteria in the vaginal niche using quantitative PCR targeting two *Megasphaera* phylotypes in a cohort of 41 women. The subjects were diagnosed by vaginal Gram stains and Amsel's criteria. Primers specifically targeting each type were tested for cross-reactivity using vaginal clones. *Megasphaera* type 1 was detected in 76% of the subjects while *Megasphaera* type 2 was found in 52% of the subjects. Moreover, *Megasphaera* type 1 concentrations were higher in subjects with BV (up to 5 orders of magnitude) than subjects without BV, and this bacterium was significantly associated with BV (*P* = .0072), as was *Megasphaera* type 2 (*P* = .0366). Phylogenetic analysis of sequence data indicated that the *Megasphaera* phylotypes form two well-supported clades that do not match sequences originating from the rumen, gut, or oral environments, suggesting that these two phylotypes may be specific to the vaginal niche.

Current treatment strategies for BV include the administration of antibiotics either orally or topically. The use of oral metronidazole for 7 days or vaginal metronidazole for 5 days results in an improvement of symptoms in 83%–87% of women within 2 to 3 weeks [104, 105]. Similar response rates are observed with the use of vaginal clindamycin. Vaginal recolonization rates with lactobacilli are similar with both antibiotics, as defined by detection of lactobacilli on Gram stain 21–30 days after start of antibiotic treatment [106, 107]. Although there is response to antibiotics in many women, persistence or recurrence of the condition occurs in 11%–29% of women at 1 month [104, 108, 109]. Moreover, long-term recurrence rates have been shown to be greater than 70% [19, 110, 111]. Marrazzo et al. [63] investigated several risk factors for BV persistence one month after treatment, including the detection of key vaginal bacteria by species-specific PCR. Persistent BV was present in 25.8% of women at the 1-month follow-up visit as determined by Amsel's clinical criteria, also confirmed by vaginal fluid Gram stains. Taxon-specific PCRs targeting bacterial 16S rRNA genes were used to detect BVAB1, BVAB2, BVAB3, *Peptoniphilus lacrimalis*, *Megasphaera* type 2, and *Mobiluncus curtisii* at baseline and 1-month follow-up visits. Data were analyzed by presence or absence of the bacteria. *Atopobium*, *Gardnerella vaginalis*, *Megasphaera* type 1, and *Lactobacillus iners* were found in ≥96% of subjects at baseline and therefore, these bacteria were not included in the assessment of risk factors for persistence. Women with BVAB1, BVAB2, or BVAB3 at baseline were shown to have a 2–8-fold increased risk of persistent BV. Likewise, presence of *P. lacrimalis* or *Megasphaera* type 2 at baseline imparted a >3-fold increased risk of persistent BV. Other risk factors such as sexual behaviors commonly linked with persistence were also examined but were not associated with persistent BV in this study. A limitation of this approach is that the persistence data was based on qualitative detection of bacteria rather than quantitative analyses. Quantitative PCR would help determine if the bacterial levels remain unchanged during antibiotic treatment (antibiotic resistance), or if the levels decline but bacteria are not eradicated, allowing for a future relapse. Another limitation of this study is the focus on women who have sex with women. It is not clear if the same patterns will hold in heterosexual women with BV.

Oakley et al. [112] performed a systematic analysis of bacterial diversity in women with and without defined BV, incorporating data from Genbank that included publicly available 16S rRNA gene sequence data obtained from the vaginal niche. A total of 969 sequences were aligned and assigned taxonomic classifications using the Greengenes 16S rRNA gene database [113]. The sequences were further analyzed based on self-similarities rather than in comparison with an external database and classified into operational taxonomic units (OTUs) using the DOTUR software package [114] at a 97% sequence similarity cutoff, which is commonly used for species definition [115]. Indeed, subjects with BV had a much greater diversity of bacteria; at the 97% cutoff, women with BV had three times the number of OTUs (15 OTUs) when compared with subjects without BV (5 OTUs). An interesting observation made in this study was that even though there was quite a bit of variability in the bacterial species between different subjects with BV, at the phylum level, the presence of bacteria from Bacteroidetes and Actinobacteria was strongly associated with BV. The authors point out that studies assessing bacterial diversity in the vaginal niche might be underestimating the true diversity by labeling bacteria with the NCBI-based designations that lump bacteria with known species. For example, sequences classified as *Prevotella* using the NCBI classification scheme of the Greengenes classification tool actually represented 21 OTUs based on the 97% cutoff using the DOTUR analytical tool, revealing an unexpectedly high number of vaginal phylotypes or species in this genus. These different vaginal phylotypes may have different functional, metabolic, and inflammatory properties. A limitation of the study by Oakley et al. [112] is that the Greengenes NCBI classification tool used may assign different identities to the same sequence simply based on sequence length. For instance, two sequences of 100% identity but different lengths can be designated as either *Gardnerella* or *Bifidobacterium*. Similarly, two identical *Atopobium* sequences different only in sequence length can either be *Atopobium* or *Olsenella*. Sequences classified as *Bifidobacterium* in the NCBI classification scheme of the Greengenes database were classified as *Gardnerella* in the RDP database. This discrepancy highlights the larger problem of defining bacterial nomenclature, which is a continuing challenge for microbial ecologists. One way of addressing this problem is to create a database of reference sequences to which all new sequences from the same niche are submitted. This would also allow rigorous tracking of novel sequences. As we develop greater understanding of the ecology of the vaginal ecosystem, we hope that all researchers will be able to use the same taxonomic nomenclature to facilitate comparisons across studies. For example, there is a human oral microbiome database that provides cross-referenced taxonomic and genomic information for approximately 600 species (http://www.homd.org/) [116].

Zhou et al. [56] studied vaginal bacterial communities in Caucasian and African American women in the United States . They applied T-RFLP analysis to 144 women ranging equally in ages and racial groups from various locations in the US. The subjects were classified as healthy based on examinations by medical personnel, but again BV status was not reported using either Amsel clinical criteria or Gram stain assessment of vaginal fluid. Restriction fragment pattern analysis resulted in the identification of 12 bacterial communities present in at least 2 women, and 8 communities present in single subjects. Using broad range 16S rRNA gene PCR primers 8f (27f-CC) and RD1r [117], 57 clone libraries were analyzed and ∼6000 clones were sequenced. Phylogenetic analysis of the 16S rRNA gene sequences obtained led to the classification of the bacterial biota into 8 “supergroups.” Five of the 8 supergroups were dominated by lactobacilli, representing 80% of the women sampled. Supergroup III, accounting for 16.5% of women sampled, had low levels of lactobacilli and a diversity of bacteria that multiple other groups have associated with BV, such as *Atopobium vaginae*, bacteria from the *Clostridiales* order, *Megasphaera*, *Dialister*, *Anaerococcus*, *Finegoldia*, *Peptostreptcoccus*, and *Eubacterium*. Since objective criteria were not used to assess for BV status (or were not reported), it is unclear if these subjects had BV or whether BV-associated bacteria colonized women without BV in this study. The study authors analyzed whether the bacterial community “supergroups” were associated specifically with race. Statistical analysis showed that supergroups III and VIII (containing a single clade of *Lachnospiraceae*) were found more often in African American women. Vaginal bacterial communities not dominated by lactobacilli were found in 33% of African American women and 7% of Caucasian women. It is known that African American women have a higher rate of BV than Caucasian women [11]. The racial differences in vaginal microbiota of “healthy” women noted in this study may simply reflect the failure to assess for BV. A substantial fraction of women with BV are asymptomatic, therefore assessing for BV status based on self-report of symptoms, as done in this study, is unreliable. Nevertheless, the fact that African American women have a higher prevalence of BV and therefore tend to have more diverse vaginal bacterial communities begs for an explanation. Strengths of the paper include the large number of samples processed, the use of T-RFLP to screen for community types, and rigorous statistical analyses applied. Limitations are the lack of objective diagnostic criteria for BV and use of a 27f primer for broad range analysis with poor homology to some vaginal bacteria that may account for the almost negligible abundance of *Gardnerella vaginalis* detected. The meaning of bacterial community “supergroups” is diminished when key members of the vaginal bacterial community are underrepresented, though this is a problem that is shared by all studies using broad range PCR to some degree.

One study assessed the vaginal microbiota from 16 women without BV (assessed by Nugent score <4), using a PCR-based approach targeting the chaperonin-60 gene (*cpn*60) [118]. Chaperonin-60 is present in all bacteria and is required for the folding and assembly of proteins and protein complexes. Most subjects were colonized largely with lactobacilli including *Lactobacillus crispatus, Lactobacillus gasseri, Lactobacillus jensenii*, and *Lactobacillus iners*. Other sequences identified included those with similarity to *Gardnerella vaginalis*, *Porphyromonas* spp., *Megasphaera* spp, and *Chlamydophila psittaci*. This is the only study that has examined the diversity of bacteria in the vaginal niche using a different target gene. This study provides a nice corroboration of results from studies using the 16S rRNA gene as a target, wherein lactobacilli have been shown to dominate the bacterial biota in subjects without BV. The detection of *C. psittaci* as part of the normal vaginal flora is interesting and rather surprising since this Chlamydia species is considered a respiratory and zoonotic pathogen and has not been previously detected in the human vagina, though it has been detected in the ovine vagina. Using a different target gene offers a different perspective on the constituents of a microbial community. However, the limited database of *cpn60* gene sequences may hinder accurate bacterial identification and the generation of phylogenetic inferences.

## 9. PYROSEQUENCING: A HIGH THROUGHPUT SEQUENCING APPROACH

While conventional sequencing techniques have provided us with a framework, the true extent of bacterial diversity in the vaginal niche is poorly understood. Analysis of the sequence data from 100 even 1000 clones results in a library with a long tail of many phylotypes detected as singlet clones when the data is represented in rank abundance plots. Based on culture techniques, it is estimated that the density of vaginal bacteria per gram of vaginal fluid ranges up to 10^8^ colony forming units [119]. If a subject has 10^8^ bacteria/gm of vaginal fluid and 100 clones are characterized, bacteria present at 10^6^ CFU or below are less likely to be included in the analysis. Moreover, classical clone library analysis tends to provide less emphasis to the long tail of minority species [51]. In fact, the census of bacteria present at low concentrations may provide important details about genetic and functional diversity in this niche [51, 120, 121]. This is especially relevant in a syndrome such as BV where we still do not understand the pathogenesis of infection.

An alternate approach for obtaining large numbers of sequences is by using pyrosequencing technology. Pyrosequencing is a “sequencing by synthesis” method which involves taking a single strand of DNA to be sequenced and sequencing the complementary strand enzymatically while monitoring the photons generated with the addition of each base [122]. The technology was applied on a small scale level to identify isolates by analyzing the signature sequences of the V1 and V3 regions of the 16S rRNA gene in 96 well plates [123, 124]. A disadvantage of the early approach was the very short read lengths obtained (25 to 100 nucleotides long) limiting accurate phylogenetic classification.

Currently, pyrosequencing technology has been further developed and it is now possible to achieve longer reads of 250 to 300 bps in a throughput of 400 000 reads per 7.5 hour run which can generate over 100 million bases (Genome Sequencer FLX System—454 Life Sciences). The extracted DNA from the vaginal sample can be amplified using fusion broad range primers (modified with adaptor sequences) targeting the variable regions of the 16S rRNA gene. The PCR products with the adaptor sequence are attached to microscopic capture beads. Emulsion-based clonal amplification (emPCR) can create several copies of the target 16S rRNA gene sequence per bead without the need for cloning the sequences into bacteria. The beads are then transferred to a picotitre plate for sequencing. Pyrosequencing technology has been used for microbial community analysis in a variety of environments [125–130].

Sundquist et al. examined the bacterial biota in vaginal samples from 6 pregnant women in all three trimesters of pregnancy using broad range bacterial PCR with deep pyrosequencing [129]. Most of the bacterial 16S rRNA gene was amplified by PCR and portions of the gene were then subjected to pyrosequencing. A total of 100 000 to 200 000 sequence reads of about 100 bp average length were obtained for each of the 6 samples. Each read was processed using the BLAT tool, a BLAST-like alignment tool [131], and a database of bacterial sequences obtained from RDP and archaeal sequences from *prokMSA.* Two major roadblocks were faced by the study investigators. First, the short read lengths made it challenging to assign phylogeny to the sequence reads. For example, while 90% of the reads were identified to the domain level, less than 10% were identified to the species level. Only about 50% of sequences were unambiguously assigned to the class level, and this was likely due to the amplification of both conserved and variable regions of the 16S rRNA gene, limiting phylogenetic resolution. The authors also performed simulation calculations and showed that increasing sequence read lengths up to 800 bp had significant impacts on phylogenetic assignments. Current 454 technology allows a read length of 250 bp with 400 bp reads on the horizon or in place at the time of this review. The second challenge encountered by the investigators was the lack of sequences in public databases resulting in many bacteria being classified as “unknown.” This problem will improve with time as more sequences are added to the databases. In accordance with data obtained from conventional cloning and sequencing experiments, the Sundquist study showed that subjects were largely colonized with lactobacilli, with a variety of other bacteria at lower concentrations including some such as *Comamonas*. In our hands, *Comamonas* spp. are common PCR contaminants that are typically present in water samples. As the study did not report results from negative controls such as sham DNA extractions with PCR and subsequent pyrosequencing, it is difficult to evaluate if *Comamonas* is indeed a part of the vaginal bacterial biota. It is imperative to conduct appropriate negative controls especially for pyrosequencing studies as the technique involves deep sequencing and can therefore easily pick up contaminating sequences even at low concentrations.

## 10. BACTERIAL VAGINOSIS: A BIOFILM SYNDROME?

Biofilms are strongly associated with human infections and up to 65% of infections treated by physicians in the developed world have been attributed to biofilms [132, 133]. There is emerging new evidence that biofilms are associated with BV [134] and it has been suggested that this biofilm may be critical in pathogenesis. Swidsinski et al. [134] demonstated the presence of adherent bacterial biofilms in 90% of subjects with BV while only 10% of subjects without BV exhibited a similar biofilm. Adherent biofilms were defined as lawns of bacteria that were tightly attached to the vaginal epithelial surface and contained specific bacterial groups. Biopsies collected from women with and without BV were sectioned and fixed for FISH and hybridized with a variety of bacterial rRNA-targeted probes. Typically, subjects with BV had an adherent biofilm that was primarily composed with 3 bacterial groups: *Gardnerella vaginalis* was present in 60 to 90% of the biofilm mass, *Atopobium* accounted for 1 to 40% of the biofilm mass, and lactobacilli were present between 1 to 5% in only 20% of the biopsy samples. Subjects without BV either had no biofilms with only a few lactobacilli scattered sporadically or had a loose bacterial biofilm which did not have any particular structure and was mainly composed of *Lactobacillus* species.

**Figure 7:**
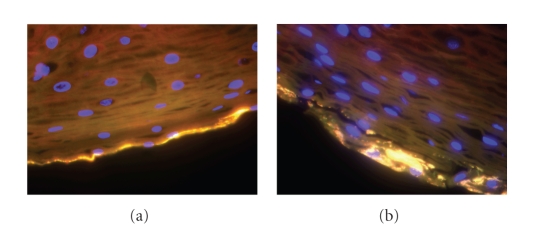
*Vaginal biopsy from a subject with BV.* A *Gardnerella vaginalis* biofilm (yellow) is detected at the edge of the vaginal epithelium (bottom) by fluorescence in situ hybridization (FISH). The yellow color is the result of using a combination of probes targeting *G. vaginalis* (Red), all bacteria (Eub338, green), and 4′,6-diamidine-2-phenylindole, dihydrochloride (DAPI, blue) which stains DNA. Note human cell nuclei in blue. The image on the right shows a vaginal epithelial cell with a cluster of *G. vaginalis* breaking off the epithelium and likely forming a clue cell.

Preliminary data from our laboratory also indicates the presence of adherent biofilms in subjects with BV (Figure 7). Biopsies obtained from women with and without BV were fixed in alcoholic formalin, sectioned and examined using FISH with a suite of bacterial rRNA-targeted probes and 4′,6-diamidino-2-phenylindole (DAPI), a DNA binding fluorescent stain. Our data also suggests the presence of a *G. vaginalis* biofilm in women with BV (Figure 7) while subjects without BV did not have a biofilm but had scattered *Lactobacillus* species.

More recently, Swidsinski et al. evaluated the effect of oral metronidazole on the BV biofilm [135]. A cohort of 18 subjects with BV, diagnosed by Gram stains and Amsel criteria, were treated with oral metronidazole for 1 week. Subsequently, follow up assessments were conducted at 1-week intervals for 5 weeks, with 3 subjects representing each point in time. Vaginal biopsies were examined using FISH probes targeting all bacteria or specific bacteria such as *Gardnerella vaginalis*, *Atopobium*, *Lactobacillus* spp., *Bacteroides/Prevotella*, and *Enterobacteriaceae*. Although, all subjects studied were considered cured of BV at the end of the antibiotic therapy, vaginal biopsies revealed a persistent biofilm. During antibiotic therapy, the biofilm could be visualized with DAPI (a DNA stain) but had poor uptake of FISH probes targeting rRNA suggesting that the bacteria were not actively metabolizing. However, at the end of 5 weeks, an actively metabolizing adherent bacterial biofilm was detected which primarily consisted of *G. vaginalis* and *Atopobium* sp. [135]. Clinically, recurrence of BV was not documented due to the limited follow-up time in the study. Important limitations of this study, also noted by the authors, include the small sample size and lack of baseline data. Furthermore, the dataset was treated as a longitudinal cohort but each time point represented a group of 3 different subjects. Despite these limitations, this study represents a novel approach to understanding the pathogenesis of BV.

Bacteria in biofilms respond differently to antibiotic treatment when compared with their planktonic counterparts [132, 136–138], and antibiotic resistance is postulated as one of the reasons for persistent and recurrent BV. A study has shown that planktonic *Gardnerella vaginalis* are more sensitive to hydrogen peroxide (5-fold) and lactic acid (4–8-fold) than *G. vaginalis* biofilm bacteria, highlighting the physiological differences that exist in the same organism under different growth conditions [139]. Several explanations are provided in the literature for the tolerance to antimicrobials by biofilm bacteria including reduced penetration of the antimicrobials within the biofilm and alterations in the stress physiology of the biofilm bacteria (reviewed in [140]). In order to circumvent issues of antibiotic resistance in bacterial biofilms, one study has used a probiotic approach to attempt clearance of the *G. vaginalis* biofilm [141]. *G. vaginalis* biofilms grown in vitro were displaced with *Lactobacillus reuteri* RC-14 and to a limited extent with *Lactobacillus iners*, commonly found in the vaginal niche. Future studies evaluating the structure and composition of biofilms in BV will become critical in understanding the pathogenesis of this common condition.

## 11. BEYOND KOCH'S POSTULATES: MOLECULAR GUIDELINES FOR CAUSATION

Robert Koch and his students elaborated a series of postulates to determine which microbes caused diseases and which microbes were colonizers without a direct etiological role (Table 1, Koch's postulates). The birth of modern microbiology in the latter half of the 19th century necessitated a system to gauge evidence of causation concordant with the discovery of numerous human and animal associated microbes through laboratory propagation. These guidelines, later called Koch's postulates, are elaborated in Koch's paper “On the Etiology of Tuberculosis” where he beautifully lays out the foundation for his thinking. Robert Koch was a prescient giant of microbiology whose thinking has served us well through more than a century of use. However, the power of Koch's postulates arises not from their rigid application, but from the spirit of critical judgment that they foster.

The esteemed researcher Edward Rosenow provided evidence that a streptococcus was the cause of poliomyelitis by fulfilling Koch's postulates [142–144], only to have this theory overturned with the discovery of poliovirus decades later. The lack of specificity demonstrated by Rosenow's false attribution of causation to streptococci in the case of polio highlights only one of many possible limitations of Koch's postulates that have emerged after more than a century of reflection (Table 2). These limitations do not seriously undermine the generally highly specific ability of Koch's postulates to identify true pathogens. If a pathogen fulfills Koch's postulates then it is most likely the cause of the disease, though these results need to be reproducible and consistent. In the case of *Gardnerella vaginalis* and BV, the ability of a pure culture of *G. vaginalis* to produce BV in 1 of 13 inoculated subjects is not a very compelling argument for causation without a better explanation for the 92% failure rate (see Section 4). Taken to its logical extreme, the successful induction of AIDS in 1 of 1000 subjects inoculated with *Mycoplasma* would also not “fulfill” Koch's third postulate for the role of *Mycoplasma* in AIDS in any meaningful or rigorous fashion. Nevertheless, the experimental reproduction of disease using pure cultures of microbes is the most powerful single approach for establishing a causal connection between a microbe and a disease. On the other hand, the failure to fulfill Koch's third postulate does not mean that a microbe is not the cause of a disease. Koch's postulates have excellent specificity for causation, but poor sensitivity. For example, many microbes have not been successfully propagated in pure culture in the laboratory; these microbes cannot fulfill Koch's postulates as originally defined. The historical evolution in thinking about causation and Koch's postulates is described elsewhere [1, 2].

## 12. A MOLECULAR VERSION OF KOCH'S POSTULATES

A major limitation of Koch's postulates is the failure to account for the possibility that uncultivated microbes play a role in disease. The use of molecular methods to characterize microbial diversity in many niches has revealed that cultivated species constitute a minority of microbes in many ecosystems, including in the human body. Many potential pathogens can be readily detected using molecular methods such as PCR. Koch's postulates can be directly translated into molecular versions, as follows.

1. The etiologic microbe or its nucleic acid sequences should be found in every case of disease. This implies that the microbe (or its products) is a sensitive indicator of disease.
2. The etiologic microbe or its nucleic acid sequences should not be found in subjects without disease. This implies that the microbe is a specific indicator of disease.
3. Experimental manipulation of infection through factors such as antimicrobial agents or induction of immune responses should demonstrate that changes in levels of an etiologic microbe correlate with disease state in the host.

## 13. DISEASE BY MICROBIAL COMMUNITY

There are some disease syndromes that may be caused by consortia of microbes rather than single pathogens. Examples of these polymicrobial syndromes are gingivitis, periodontitis, and BV. Proving that a single cultivated or uncultivated microbe is the cause of a disease can be challenging. Proving that a microbial consortium is the cause of a disease is even more daunting.

Microbes probably exist in communities in order to take advantage of syntrophic relationships wherein the metabolic end product of one species is the energy source for a second species. If critical members of the community are lost, then the metabolic networks collapse and all members of the community may suffer. However, functional redundancy among microbes may mean that bacterium A is not necessary for community health as long as bacterium B is present with its overlapping metabolic capacity. What does this mean if bacteria A and B are part of a pathogenic community? It means that neither bacterium will be deemed necessary for disease, because subjects may have disease when lacking bacterium A or B, though subjects will not have disease if lacking both bacteria. Bacteria A and B are considered sufficient when part of the larger community, but not individually necessary for establishing the community and producing condition. To address this issue, we will need to assess not only the species composition of pathogenic microbial communities, but also the metabolic capabilities and interdependencies of these communities. Studies of the human microbiome will be vital in filling this knowledge gap.

## 14. CONCLUSIONS

In the last two decades, there has been a dramatic increase in our understanding of the bacterial biota in a variety of ecological environments using cultivation-independent molecular methods. These methods have recently been applied to the human vaginal microbial ecosystem, adding substantial data on bacterial diversity in this niche. Subjects without BV have bacterial biotas that are less complex and are dominated by *Lactobacillus* species. Subjects with BV have loss of *Lactobacillus crispatus* and acquisition of more complex vaginal bacterial communities that include many heretofore-uncultivated species. Data emerging from molecular investigations suggest that it is possible to develop a PCR-based strategy for the diagnosis for BV. BV may be an example of a condition produced by a pathogenic microbial community rather than a single pathogen, presenting many challenges for understanding the etiology and pathogenesis of this syndrome. A molecular version of Koch's postulates is presented for collecting evidence of causation for uncultivated microbes such as those linked to BV. There is new evidence suggesting that BV may be a biofilm condition in some women, which may contribute to poor treatment responses and high relapse rates. Understanding the bacterial biota of the human vagina is critical for optimizing reproductive health, and although many advances have been made, there is much that is unknown about how bacterial communities in the human vagina promote health and facilitate disease.

## Figures and Tables

**Table 1:** Koch's postulates [1].

The etiologic microbe should be found in every case of the disease
The etiologic microbe should not be found in subjects without disease (specificity)
The etiologic microbe should be isolated in pure culture on lifeless media and be capable of causing the characteristic disease anew upon inoculation in a susceptible host
The etiologic microbe should be reisolated from the experimentally inoculated host.

**Table 2:** Limitations of Koch's postulates.

Ignore the contribution of host, vector, and environment to disease susceptibility/response
Colonization state (e.g., +PPD skin test for tuberculosis in the absence of disease) violates Koch's second postulate
Many pathogens cannot be propagated on lifeless (cell-free) medium in the lab; these pathogens cannot fulfill Koch's third postulate
Viruses, parasites, uncultivated bacteria may not grow in pure culture
Host range restriction of pathogens
Do not consider the possibility of disease produced by a microbial community rather than a single pathogen
Not completely specific
